# The impact of escitalopram on vagally mediated cardiovascular function to stress and the moderating effects of vigorous physical activity: a randomized controlled treatment study in healthy participants

**DOI:** 10.3389/fphys.2013.00259

**Published:** 2013-09-24

**Authors:** Camilla S. Hanson, Tim Outhred, Andre R. Brunoni, Gin S. Malhi, Andrew H. Kemp

**Affiliations:** ^1^SCAN Research and Teaching Unit, School of Psychology, University of SydneySydney, NSW, Australia; ^2^Discipline of Psychiatry, Sydney Medical School, Northern Clinical School, University of SydneySydney, NSW, Australia; ^3^CADE Clinic, Royal North Shore HospitalSt. Leonards, NSW, Australia; ^4^Center for Clinical and Epidemiologic Research, University Hospital, University of São PauloSão Paulo, Brazil

**Keywords:** selective serotonin reuptake inhibitors (SSRIs), escitalopram, exercise, physical activity, cardiovascular stress response, heart rate, heart rate variability, HRV

## Abstract

Recent concerns over the impact of antidepressant medications, including the selective serotonin reuptake inhibitors (SSRIs), on cardiovascular function highlight the importance of research on the moderating effects of specific lifestyle factors such as physical activity. Studies in affective neuroscience have demonstrated robust acute effects of SSRIs, yet the impact of SSRIs on cardiovascular stress responses and the moderating effects of physical activity remain to be determined. This was the goal of the present study, which involved a double-blind, randomized, placebo-controlled, cross-over trial of a single-dose of escitalopram (20 mg) in 44 healthy females; outcomes were heart rate (HR) and its variability. Participants engaging in at least 30 min of vigorous physical activity at least 3 times per week (regular exercisers) showed a more resilient cardiovascular stress response than irregular vigorous exercisers, a finding associated with a moderate effect size (Cohen's *d* = 0.48). Escitalopram attenuated the cardiovascular stress response in irregular exercisers only (HR decreased: Cohen's *d* = 0.80; HR variability increased: Cohen's *d* = 0.33). HR during stress under escitalopram in the irregular exercisers was similar to that during stress under placebo in regular exercisers. These findings highlight that the effects of regular vigorous exercise during stress are comparable to the effects of an acute dose of escitalopram, highlighting the beneficial effects of this particular antidepressant in irregular exercisers. Given that antidepressant drugs alone do not seem to protect patients from cardiovascular disease (CVD), longitudinal studies are needed to evaluate the impact of exercise on cardiovascular stress responses in patients receiving long-term antidepressant treatment.

## Introduction

Depression and cardiovascular disease (CVD) are leading burdens of disease and this burden is projected to worsen up to 2030 and beyond with ageing of the population and increasing prevalence of multi-morbidity (Mathers and Loncar, 2006; Langan et al., 2013). Critically, depression increases risk for the development of CVD 1.5-fold, while patients with CVD and depression have a 2- to 3-fold increased risk of future cardiac events compared to those cardiac patients who do not have depression (Rudisch and Nemeroff, 2003). Psychological stress plays a key role in the development of depression (Cohen et al., 2007), and increases risk of mortality from a number of causes including CVD, cancer and external causes over an 8 year follow-up period (Russ et al., 2012). While many biological factors, including the hypothalamic-pituitary-adrenal (HPA) axis dysfunction and altered inflammatory processes, contribute to the relationship between stress, psychiatric illness and CVD, vagally mediated cardiovascular function—indexed by increases in heart rate (HR) and decreases in heart rate variability (HRV)—may underlie a substantial part of this risk (Thayer et al., 2010a; Nemeroff and Goldschmidt-Clermont, 2012). Importantly, the inhibitory function of the vagus nerve regulates both the HPA axis and inflammatory processes (Tracey, 2002; Thayer and Sternberg, 2006; Huston and Tracey, 2010; Kemp and Quintana, 2013), leading to proposals that chronic impairment in vagal function is an early marker of future morbidity and mortality (Kemp et al., 2010; Thayer et al., 2010b; Åberg et al., 2013; Kemp and Quintana, 2013).

Acute psychological stress is associated with parasympathetic withdrawal, sympathetic activation, an increase in HR and reductions in its variability (Madden and Savard, 1995; Porges, 1995; Steptoe and Kivimäki, 2012), while chronic stress is associated with persistent cardiovascular stress responses, which may then contribute to psychiatric illness, physical ill-health and all-cause mortality (Thayer and Brosschot, 2005; Thayer et al., 2010b; Lemogne et al., 2011; Åberg et al., 2013; Kemp and Quintana, 2013). Epidemiological research has even demonstrated that an increased resting HR increases the risk of suicide by 19–37% over a follow-up period of 9 years even after accounting for covariates including depressed mood (Lemogne et al., 2011). In another study lower cardiovascular fitness determined using the cycle ergonometric test at 18 years of age predicts an increased risk of suicide attempt and death by suicide over a follow-up period of 42 years (Åberg et al., 2013). As lower cardiovascular fitness is associated with increased HR and reduced HR variability (Rennie, 2003), these findings highlight an important role of vagally mediated cardiovascular function in regards to psychological wellbeing as well as physical health. Importantly, increased parasympathetic control is associated with positive emotions (Kok and Fredrickson, 2010), resilience (Kashdan and Rottenberg, 2010) and improved regulation over the HPA axis and inflammatory processes (Tracey, 2002; Thayer and Sternberg, 2006; Huston and Tracey, 2010; Kemp and Quintana, 2013), thereby contributing to psychological wellbeing and physical health. National guidelines (Australian Government Department of Health and Ageing: http://www.health.gov.au/internet/main/publishing.nsf/content/health-pubhlth-strateg-phys-act-guidelines) distinguish between regular moderate-intensity physical activity and regular vigorous exercise, recommending at least 30 min of vigorous exercise on top of regular activity for health and fitness benefits.

The selective serotonin reuptake inhibitors (SSRIs) are a first-line treatment option (Kemp et al., 2008) for both mood and anxiety disorders and admixtures of the two, and are also used widely in bipolar depression (Malhi, 2012). SSRIS have important acute neurophysiological effects (Kemp and Nathan, 2004; Kemp et al., 2004) providing the foundation upon which subsequent therapeutic response is based (Harmer et al., 2009). Acute effects are often studied in healthy samples to avoid confounds of illness and psychopathology (Harmer, 2010). Interestingly, acute SSRI treatment has been shown to attenuate cardiovascular responses to stress (Straneva-Meuse, 2004a; Golding et al., 2005; Jiang et al., 2013), However, longitudinal research indicates that they may also have adverse cardiovascular effects, including reductions in vagally mediated cardiovascular function (Licht et al., 2010b) and sudden cardiac death (Whang et al., 2009), highlighting the importance of research on antidepressant actions and the moderating effects of specific lifestyle factors such as physical activity. Physical activity is associated with improved psychological health (Powell et al., 2011; Åberg et al., 2013) and lower cardiovascular (and proinflammatory) responses to stress (Hamer and Steptoe, 2007) lowering the risk of future CVD (Mora et al., 2007; Hamer and Stamatakis, 2009; Hamer et al., 2009) and mortality (Leitzmann et al., 2007). The higher the parasympathetic activity at rest—indicated by lower HR and higher HRV—the more autonomic resources available to tackle subsequent stressors; a proposal known as the “autonomic resource hypothesis” (Hynynen et al., 2008; Boullosa et al., 2012). Thus, research on the impact of antidepressants and the moderating effects of physical activity has important implications for not only better understanding therapeutic response to antidepressant medications, but also for the long-term health of the cardiovascular system. Recent research in outpatients with coronary heart disease and depression (Blumenthal et al., 2012) demonstrates that exercise and sertraline (a SSRI) are equally effective at reducing depressive symptoms after 16 weeks of treatment, but that exercise led to greater improvements in HRV collected using 24-h Holter recordings compared with sertraline. However, it remains unclear, to what extent physical activity and SSRIs interact in moderating the effects of SSRIs on psychological stress.

Here we report on a proof-of-concept, laboratory-based, experimental study to examine the acute effects of a commonly prescribed SSRI, escitalopram, on HR and its variability under stress, with the aim of determining whether individual differences in levels of physical activity moderate these effects. Better understanding the impact of the SSRIs and the moderating effects that specific lifestyle factors exert has important implications for therapeutic responsiveness, psychological wellbeing, and physical health. We focus specifically on acute treatment effects in healthy female volunteers. We hypothesized that acute administration of escitalopram would attenuate increases in HR and decreases in HRV during stress, relative to placebo, and that regular vigorous exercise would facilitate this effect.

## Methods

### Participants

Forty-four healthy female volunteers (aged 18–47, *M* = 23.70, *SD* = 5.89) completed the study, and gave written informed consent in accordance with National Health Medical Research Council guidelines. Participants were recruited using university-wide staff and student newsletters. Participants were free from medication (other than hormonal contraceptives), physical and psychiatric illness, symptoms of depression and anxiety [PHQ-9 (Kroenke et al., 2001) and GAD-7 (Spitzer et al., 2006) assessment], illicit drug use, alcoholism, smoking, brain injury, neurological disorders, and sustained loss of consciousness. Only female participants were recruited for this study as females display higher rates of mental disorders (Nolen-Hoeksema, 2001; Kessler, 2003; Slade et al., 2009); focusing on females also allowed us to avoid known gender differences in baseline HRV (Rajendra Acharya et al., 2006), HRV responses to cognitive stress (Li et al., 2009), and responsiveness to SSRI treatment (Khan et al., 2005). Finally, participants abstained from caffeine on the morning of the experiment and no participant tested positive on pregnancy tests, which were conducted at each session. Ethics approval for the trial was secured from the University of Sydney's Human Research Ethics Committee (ref. 13901) and the Northern Sydney Central Coast Area Health Service Human Research Ethics Committee (ref. 1105-178 M), and it was registered with the Australian New Zealand Clinical Trials Registry (ANZCTR; ACTRN126111000719932).

### Experimental design

Participants were randomly assigned to receive escitalopram (20 mg) or placebo *per os*, tested in a double-blind crossover design, with two sessions per participant separated by at least 1 week to ensure a sufficient drug washout of at least 5 half-lives (*t*_1/2_ = 26.7 h; Sogaard, 2005). Forty-five percent of participants received placebo at the first session. Relative to other commonly prescribed SSRIs, escitalopram has improved efficacy, a lower side effect profile, and greater selectivity for serotonergic transport proteins (Fernandez et al., 2007; Cipriani et al., 2009a). The maximum recommended dosage of 20 mg was used to maximize receptor occupancy (Kasper et al., 2009) and subsequent physiological effects from a single dose.

### Measurement of physical activity

Participants reported the frequency in which they engaged in vigorous physical activity using the International Physical Activity Questionnaire (IPAQ; Craig et al., 2003), a widely used, reliable and valid self-report assessment. Physical activity data is transformed into energy expenditure estimates of metabolic equivalent tasks (MET), such that one MET is equivalent to the energy cost of sitting quietly for an hour (1 kcal/kg/h). Participants were categorized into low or high vigorous activity groups—according to national guidelines (Australian Government Department of Health and Ageing: http://www.health.gov.au/internet/main/publishing.nsf/content/health-pubhlth-strateg-phys-act-guidelines). These guidelines recommend at least 30 min of vigorous physical activity 3 days a week on top of regular moderate-intensity activity for health and fitness benefits. Participants categorized in the low activity grouping did not meet the criteria of at least 30 min of vigorous physical activity 3 days a week, while participants in the high activity grouping met or exceeded this criterion. Groups did not differ on time spent sitting, walking or performing moderate physical activity (all *p* > 0.1). Groups were compared on resting state HR, providing a (partial) validation of questionnaire-based categorization (see participant characteristics).

### Stress manipulation

The mental arithmetic task component of the Trier Social Stress Test (TSST; Kirschbaum et al., 1993) was used to elicit stress (Jönsson et al., 2010) and associated physiological correlates, including HR, blood pressure, catecholamines and cortisol (Straneva-Meuse, 2004a; Vermetten, 2006). Participants were instructed to count backwards subtracting thirteens, beginning at either 2083 or 2027 for 5-min. Participants were given one of these two alternate versions of the arithmetic task across sessions. No feedback was given for correct responses, and the experimenter vocalized the word “error” when a mistake was made, instructing the participant to start counting from the beginning, further increasing stress and social pressure as per prior studies (Hjortskov et al., 2004).

### Procedure

Testing was conducted in a psychophysiology laboratory at the Clinical Assessment and Diagnostic Evaluation (CADE) Clinic (www.cadeclinic.com), Royal North Shore Hospital. Participants arrived in the early morning, having consumed breakfast, and abstained from caffeine, as caffeine increases sympathetic nervous system (SNS) activity (Sondermeijer et al., 2002). Participants completed a consent form and pregnancy test. Height and weight were measured at the first session to calculate BMI. Testing commenced 3 h after administration of either placebo or 20 mg of escitalopram, so as to coincide with the time of the expected peak plasma concentration (*T*_max_ = 3.0 ± 1.5 h; Sogaard, 2005; Rao, 2007) During the waiting period, the participant was provided with a standardized snack and in the first session only, completed the IPAQ. The snack helped to minimize autonomic and mood changes associated with hunger. In order to determine the presence of side effects, subjective wellness was recorded during the waiting period and after psychophysiological testing. Participants reported whether they experienced side effects and were asked to guess their treatment condition in order to examine the potential impact of treatment unblinding at each session.

All participants were tested in the late morning, and at the same time on each session to control for potential changes in HRV with circadian rhythms (Kleiger et al., 2005). Participants sat in a reclined chair with their legs raised and were instructed to breathe normally, remain still and awake for 5-min of data collection in the resting state. Following this, 5-min recordings were made during the stress condition in which participants were required to perform the mental arithmetic task. The experimenter was seated in close proximity to the participant and acted in a formal manner, in order to further increase the stressful nature of the procedure through social pressure (Hjortskov et al., 2004). After testing, subjective ratings of relaxation and stress for each condition were recorded on a five-point Likert scale (from low to high) to determine the efficacy of the experimental manipulations.

Participants provided saliva samples (1 mL) for estradiol and progesterone analysis before treatment administration at each session. The hormonal saliva samples were stored frozen until assay. On the day of assay, samples were thawed for determination of salivary progesterone and estradiol using commercially available kits (Salimetrics, USA) according to the manufacturers instructions. Thawed samples were centrifuged at 1500 × *g* for 15 min to collect clear saliva which was used without further processing for all assays. All samples were brought to room temperature before adding to assay wells and all samples were analyzed in duplicate. Hormonal menstrual phase was determined in accordance with previous research (Lu et al., 1999; Gandara et al., 2007).

### Data collection and analysis

HR and R-R interval recordings—on which analyses of HRV were conducted—were made during two conditions, consistent with Task Force guidelines (Task Force of the European Society of Cardiology and the North American Society of Pacing and Electrophysiology, 1996): (1) a 5-min resting state and (2) a 5-min social stress task, using a Polar RS800CX training device at a sampling rate of 1000 Hz. These devices have been validated against the electrocardiogram (ECG) and have excellent reliability, especially in young, healthy individuals in a supine position and when analysis is conducted on normalized values (Gamelin et al., 2006; Weippert et al., 2010) (see also Quintana et al., 2012), as was the case in the current study. This device is wirelessly connected to an electrode strap worn just beneath the chest, that has been wetted with saline solution (0.9% NaCl) to simulate sweat and ensure conductivity. R-R intervals were extracted from text files and analyzed in Kubios HRV analysis software (version 2.0, 2008, biosignal analysis and medical imaging group, University of Kuopio, Finland, MATLAB). Each file was visually inspected for artifacts (ectopic and missing beats), and medium automatic filter corrections were applied to each data set. Measures of HRV included HF HRV (normalized units, n.u.) (0.15–0.40 Hz) and the Root Mean Square of the Successive Differences (RMSSD).

### Statistical analysis

All statistical analyses were conducted using IBM SPSS Statistics (Version 20) for Windows 7. The impact of escitalopram and the moderating effects of physical activity levels on HR and heart rate variability (HRV) were examined using mixed within- and between-subjects analysis of variance (ANOVA). Within subject factors were treatment (escitalopram vs. placebo) and task (stress vs. rest) and the between subjects factor was physical activity (high vs. low). A factor of “treatment order” was not included in statistical analysis for parsimony; order effects are rare, are generally underpowered even when an appreciable effect is present, and sufficient drug washout ameliorates such concerns (Senn, 1994; Senn et al., 2004; Mills et al., 2009). Independent samples *t*-tests were conducted to determine whether low and high activity groupings differed across demographic variables (Table 1). ANOVA was also employed to determine whether subjective ratings of relaxation and stress differed across treatment sessions and physical activity groupings. Cohen's *d* effect size statistics were calculated for each pairwise comparison. Cohen's guidelines (Cohen, 1988a,b) identify 0.2, 0.5, and 0.8 as small, medium, and large effects, respectively. Partial eta-squared (η^2^_*p*_) was reported for ANOVA effects as an indicator of effect size (small = 0.01 medium = 0.06, large = 0.14; (Cohen, 1988a,b). The statistical threshold of 0.05 (two-tailed) was set for all analyses, except where directional effects were expected, in which one-tailed tests were employed. Manipulation checks included a focus on whether hormone or menstrual status, or treatment blinding differentially impacted on the results reported below. With respects to hormone and menstrual status, χ^2^ tests were conducted to check for equal proportions of participant menstrual phase within treatment sessions and equal proportions of participants using hormonal contraceptives in each order of treatment. Regression analyses were also conducted on hormone concentrations in order to determine the relationships between hormone levels and the HR and HRV responses under each treatment and task condition. With respects to treatment blinding, χ^2^ tests were performed to examine the relationship between guessing treatment condition and experiencing side effects. The effect of guessing and side effects on HR and HRV under each treatment and task condition was determined using repeated-measures ANOVAs on HR and HRV separately.

**Table 1:** **Participant demographic information**.

	**Low PA (*n* = 18)**		**High PA (*n* = 22)**		**Statistics**
	**Mean**	***SD***	**Mean**	***SD***	
Age (years)	24.06	7.78	23.41	3.91	*t*_(38)_ =0.34, *p* = 0.735
Education (years)	16.11	2.76	16.77	2.83	*t*_(38)_ = −0.74, *p* = 0.462
Ethnicity (C/NC)	1/17		1/21		χ^2^_(1)_ = 0.021, *p* =0.884
BMI (kg/m^2^)	21.88	2.60	23.40	3.30	*t*_(38)_ = −1.58, *p* = 0.122
Hormonal contraceptive use (Y/N)	9/9		12/10		χ^2^_(1)_ = 0.082, *p* = 0.775
Menstrual phase placebo (F/M/L)	8/5/5		8/6/8		χ^2^_(2)_ = 0.387, *p* = 0.824
Menstrual phase drug (F/M/L)	6/5/7		10/8/4		χ^2^_(2)_ = 2.132, *p* = 0.344
Side effects (Y/N)	8/10		11/11		χ^2^_(1)_ = 0.123, *p* = 0.726
Correct treatment guess (Y/N)	14/4		14/8		χ^2^_(1)_ = 0.943, *p* = 0.332
AUDIT total (SD)	6.11 (4.13)		6.45 (5.32)		*t*_(38)_ = −0.224, *p* = 0.824
Smoking (Y/N)	0/18		0/22		n/a
PHQ-9 (SD)	1.61 (1.20)		1.45 (1.22)		*t*_(38)_ = 0.407, *p* = 0.686
GAD-7 (SD)	0.72 (0.895)		1.41 (1.26)		*t*_(38)_ = −1.945, *p* = 0.059
IPAQ (mins/wk)					
Vigorous intensity activity	30.28	58.37	207.05	94.30	*t*_(38)_ = −6.93, *p* < 0.001 (one-tailed), *d* = 2.26
Moderate intensity activity	206.11	230.12	278.41	222.43	*t*_(38)_ = −1.01, *p* = 0.320
Walking	461.94	297.85	457.50	284.12	*t*_(38)_ = 0.05, *p* = 0.962
Sitting	366.67	180.75	351.82	153.30	*t*_(38)_ = 0.28, *p* = 0.780
Total physical activity	698.33	428.23	942.94	408.14	*t*_(38)_ = −1.85, *p* = 0.073
Energy expenditure, METs-min/week	2620.53	1650.94	4546.11	1793.27	*t*_(38)_ = −3.50, *p* = 0.001, *d* = 1.12
Resting HR	71.78	8.56	66.14	14.10	*t*_(38)_ = 1.71, *p* = 0.048 (one-tailed), *d* = 0.55

Abbreviations *PA* *physical activity* *BMI* *body mass index* *PHQ-9* *patient health questionnaire* *GAD-7* *generalised anxiety disorder screener* *IPAQ* *international physical activity questionnaire* *MET* *metabolic equivalent task*.

## Results

### Participant characteristics

**Figure 1:**
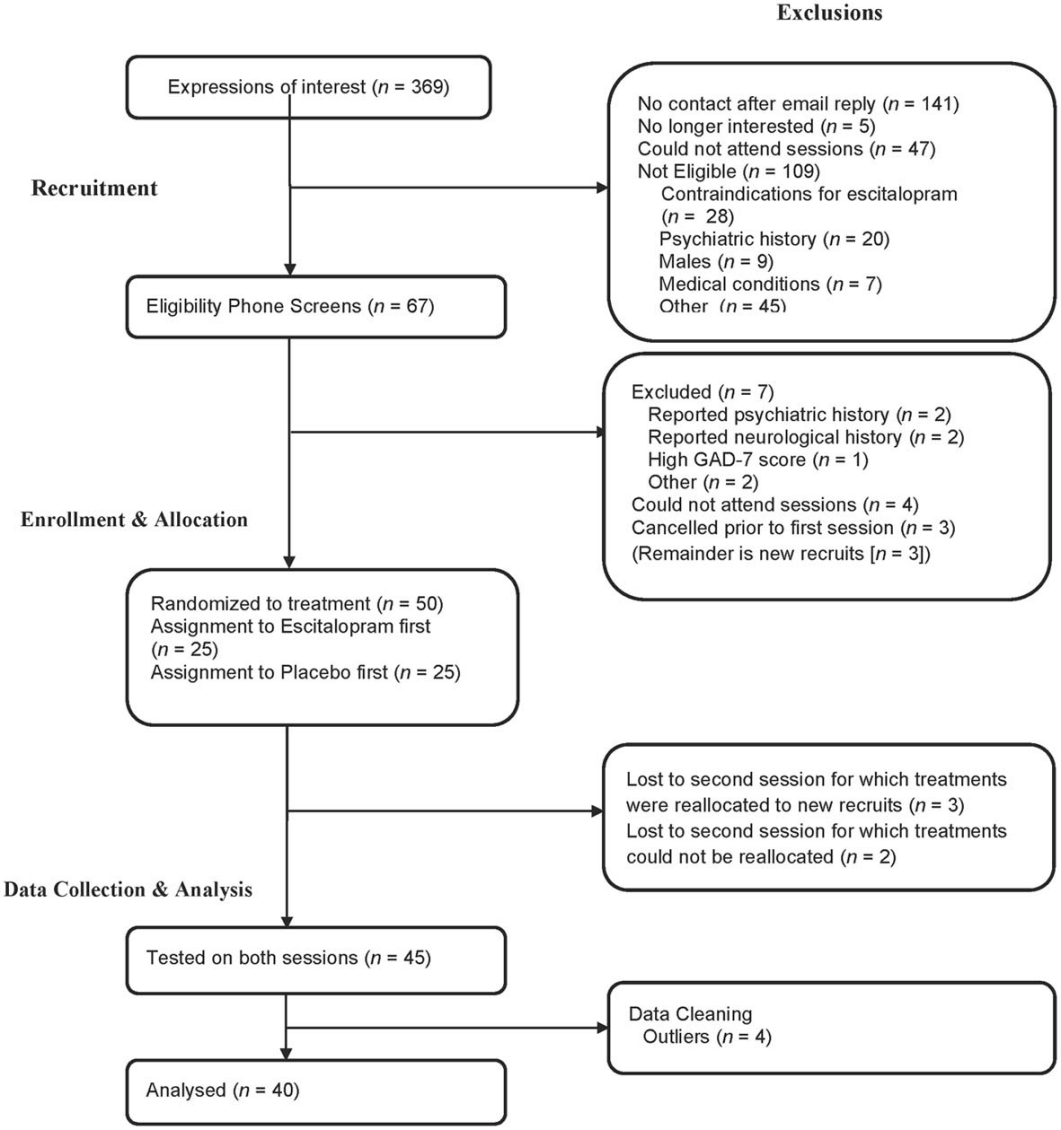
**A depiction of participants' attrition from the experiment and the reasons and stages at which they were excluded**.

Participant characteristics are presented in Table 1 and flow of participants through the different stages of the study is presented in Figure 1. The two physical activity groupings were matched in terms of age, BMI, years of education, time spent sitting, walking or performing moderate physical activity (all *p* > 0.1). The high activity group spent more minutes in the past week in vigorous activity [*t*_(38)_ = 6.93, *p* < 0.001, *d* = 2.26], and had a higher weekly energy expenditure [*t*_(38)_ = 3.50, *p* = 0.001, *d* = 1.12] relative to the low activity group. Regardless of treatment, resting HR was significantly lower in the high activity group in comparison to the low activity group [*t*_(38)_ = 1.71, *p* = 0.048 (one-tailed), *d* = 0.55], suggesting cardiovascular adaptations in the high activity group that may be explained by the link between bradycardia, regular exercise and physical fitness. Four participants were identified as multivariate outliers on HR variables using Tabachnick and Fidell's (2007) multivariate outliers identification procedure, and were subsequently excluded from further analysis. Forty participants aged between 18 and 47 (*M* = 23.70, *SD* = 5.89) were included in final analyses.

### Hormonal results

The χ^2^ tests demonstrated that participants were equally distributed between menstrual phases on placebo, χ^2^_(2)_ = 0.950, *p* = 0.622, and escitalopram, χ^2^_(2)_ = 0.950, *p* = 0.622, treatment sessions. There was an equal distribution of participants who took hormonal contraceptives to those who did not, χ^2^_(2)_ = 0.100, *p* = 0.752 and hormonal contraceptive status was equally distributed between those who received placebo or escitalopram at their first session, χ^2^_(1)_ = 0.002, *p* = 0.962. Regression analyses on HR and HRV data for each task and treatment condition showed no significant associations with progesterone or estradiol concentration [all *p* > 0.05 (Bonferroni corrected for multiple analyses)]. Additionally, there were no differences between those who did and did not take hormonal contraceptives on each task and treatment condition [HR, *F*_(1, 38)_ = 0.544, *p* = 0.465; HRV, *F*_(2, 37)_ = 0.733, *p* = 0.487].

### Treatment blinding manipulation check

A greater proportion of participants (70%) were found to correctly guess treatment condition than that which was expected by chance, χ^2^_(1)_ = 6.400, *p* = 0.011. Side effects also occurred in 48% of the sample, although these did not occur in a greater proportion than that expected by chance, χ^2^_(1)_ = 0.100, *p* = 0.752. There was, however, a significant association between the subjective experience of side effects and correct treatment guessing, χ^2^_(1)_ = 6.535, *p* = 0.011, indicating that participants who correctly guessed treatment condition were likely to have made this correct guess based on their experience of side effects. Repeated-measures ANOVAs testing the interaction between presence of side effects and correct guessing of treatment were performed on the HR and HF HRV measures. Importantly, no significant interaction effects of correct guessing × side effects × treatment × task on HR [*F*_(1, 36)_ = 0.428, *p* = 0.517] or HRV [*F*_(1, 35)_ = 0.995, *p* = 0.380] were observed.

### Subjective ratings of task

A significant interaction between task and physical activity groupings on subjective ratings was observed, [*F*_(1, 32)_ = 7.17, *p* = 0.012, η^2^_*p*_ = 0.18]. Follow-up pairwise comparisons demonstrated that participants in the high physical activity group reported significantly lower mental stress during the stress task, than the low physical activity group, *t*_(32)_ = 2.84, *p* = 0.008, *d* = 1.00. Therefore, the less physically active group found the mental stress task more stressful than the more physically active group, regardless of treatment. Importantly, however, a main effect of task was also observed indicating that participants were more stressed in the arithmetic task than during the rest condition [*F*_(1, 32)_ = 102.77, *p* < 0.001, η^2^_*p*_ = 0.76], confirming the validity of the stress condition in our sample, regardless of physical activity groupings. No main effects of physical activity [*F*_(1, 32)_ = 2.49, *p* = 0.124] or treatment [*F*_(1, 32)_ = 2.35, *p* = 0.135], or interactions between treatment and task [*F*_(1, 32)_ = 2.26, *p* = 0.142] or treatment, task, and physical activity [*F*_(1, 32)_ = 0.228, *p* = 0.637] were observed on subjective ratings.

### Impact of escitalopram and the moderating effects of physical activity

**Figure 2:**
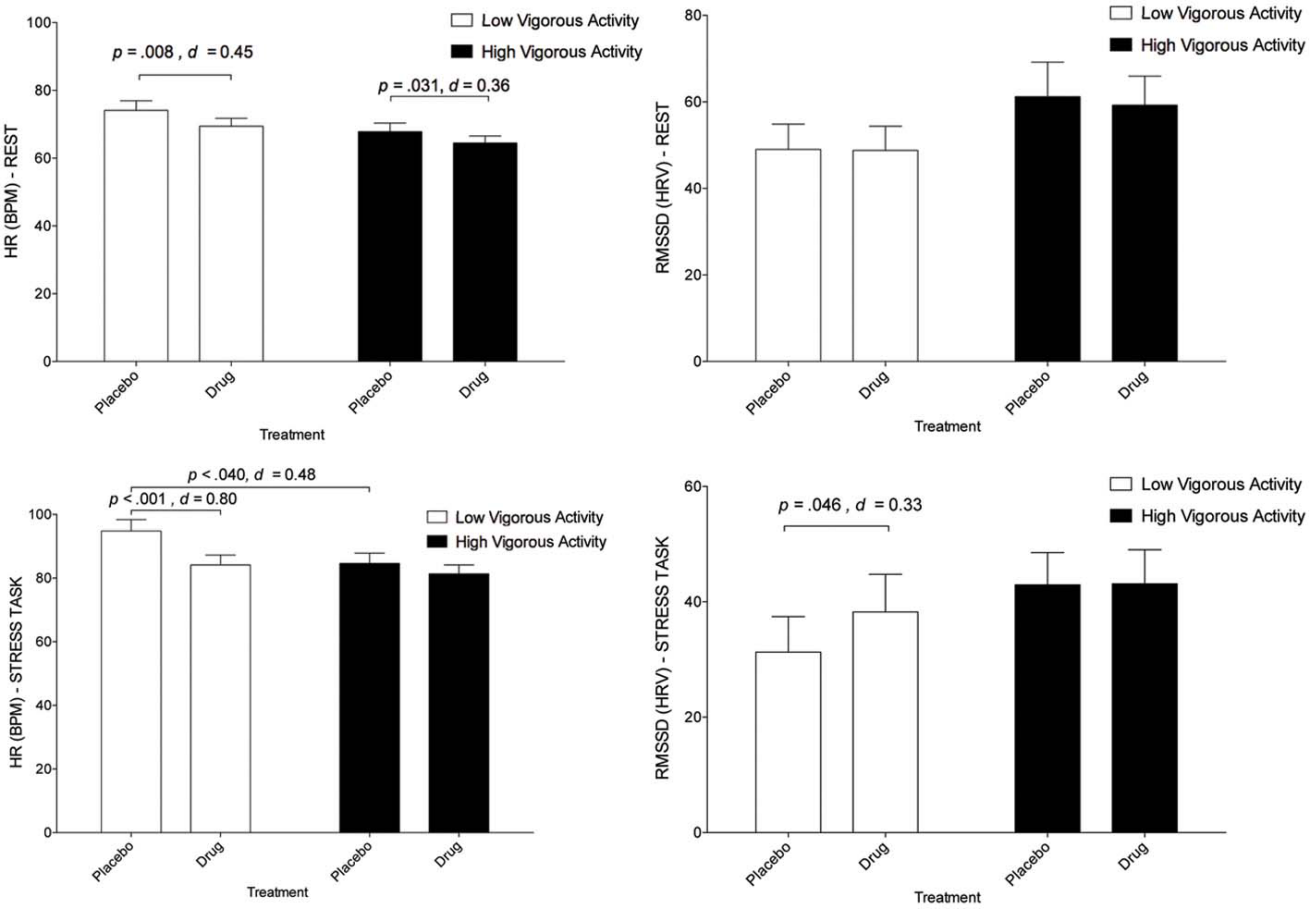
**The interaction between treatment and physical activity category on cardiovascular responses (with standard error bars).** Top left graph: Effects on HR at rest. Top right graph: effects on RMSSD at rest. Lower left graph: Effects on HR during stress. Lower right graph: effects on RMSSD during stress.

Descriptive statistics for HR and HRV in high and low activity groupings for task and treatment are presented in Figure 2 and Table 2.

**Table 2:** **HR and RMSSD means and standard deviations for vigorous exercise groups under each treatment for rest and stress conditions**.

		**Low vigorous activity**		**%Δ**	**High vigorous activity**		**%Δ**
		**Placebo *M* (*SD*)**	**Drug *M* (*SD*)**		**Placebo *M* (*SD*)**	**Drug *M* (*SD*)**	
HR	Rest	74.13 (8.56)	69.43 (6.93)	−6.3	67.84 (14.10)	64.45 (11.83)	−5
	Stress	94.80 (10.45)	84.12 (10.03)	−11.2	84.62 (17.93)	81.34 (15.21)	−3.9
	%Δ	27.9	21.2		24.7	26.2	
RMSSD	Rest	49.03 (24.80)	48.79 (23.77)	−0.4	61.22 (37.45)	59.27 (31.41)	−3.2
	Stress	31.27 (14.18)	38.25 (18.17)	22.3	42.94 (32.81)	43.13 (33.45)	0.4
	%Δ	−36.2	−21.6		−29.9	−27.2	

#### Heart rate

A three-way interaction between treatment, task and activity level was significant, [*F*_(1, 38)_ = 7.18, *p* = 0.011 η^2^_*p*_ = 0.16]. Relative to placebo, escitalopram reduced HR in both the low activity [*t*_(38)_ = 2.80, *p* = 0.008, *d* = 0.45] and high activity [*t*_(38)_ = 2.24, *p* = 0.031, *d* = 0.36] groups during rest. Escitalopram also reduced HR in the low activity group [*t*_(38)_ = 4.97, *p* < 0.001, *d* = 0.80] during stress. By contrast, escitalopram did not reduce HR in the high activity group during stress (Figure 2). Notably, HR for the high activity group during stress under placebo, was markedly reduced relative to those in the low activity group [*t*_(38)_ = 2.13, *p* = 0.040, *d* = 0.48], and equivalent to HR for the low activity group under drug (Figure 2), indicating that those in the high activity group display a more resilient cardiovascular response to stress than those in the low activity group. This interpretation is further supported by the observation of an interaction between task and physical activity grouping on subjective ratings of task (reported above). These findings were observed on a background of a significant treatment × task interaction [*F*_(1, 38)_ = 6.61, *p* = 0.014, η^2^_*p*_ = 0.15], a main effect of treatment [*F*_(1, 38)_ = 22.18, *p* < 0.001, η^2^_*p*_ = 0.37], and a main effect of task [*F*_(1, 38)_ = 210.43, *p* < 0.001, η^2^_*p*_ = 0.85] collapsed across physical activity groupings. These findings indicate that the increase in HR under stress (relative to rest) was decreased by escitalopram (a treatment × task interaction), that HR was decreased by escitalopram (a main effect of treatment) and that HR was increased under stress (a main effect of task).

#### Heart rate variability

No significant three-way interaction was found for HRV [*F*_(2, 37)_ = 0.23, *p* = 0.80]. Univariate tests for the three-way interaction between treatment, task and vigorous activity were not significant for HF *F*_(1, 38)_ = 0.06, *p* = 0.804, or RMSSD, *F*_(1, 38)_ = 0.47, *p* = 0.496. However, a main effect of task was observed indicating that HRV was decreased by stress, relative to the rest, regardless of treatment [RMSSD, *F*_(1, 38)_ = 35.99, *p* < 0.001, *d* = 0.96; and HF, *F*_(1, 38)_ = 84.60, *p* < 0.001, *d* = 1.47]. Interestingly, *post-hoc*, pairwise comparisons indicated that escitalopram—relative to placebo—increased RMSSD (but not HF) during stress in the low activity group [*t*_(37)_ = 2.07, *p* = 0.046, *d* = 0.33], mirroring the findings of HR. No other pairwise comparisons were significant.

## Discussion

Recent reports on the adverse cardiovascular effects of the second-generation antidepressants (Whang et al., 2009; Licht et al., 2010a), highlight the importance of research on specific lifestyle factors such as physical activity. Major findings observed here were that (a) participants in the high physical activity group (regular vigorous exercisers) reported feeling less stressed than those in the low physical activity grouping (irregular exercisers) during the stress task (i.e., an interaction between task and physical activity groupings on subjective ratings on task); this finding was associated with a large effect size (Cohen's *d* = 1.00), (b) all participants reported feeling more stressed in the stress vs. rest condition (i.e., a main effect of task), a finding associated with a large effect size (η^2^_*p*_ = 0.76), providing an important validation of our task in the context of an interaction between group and physical activity grouping, (c) irregular—relative to regular—exercisers display attenuated cardiovascular responses to stress following treatment with escitalopram (i.e., a three-way interaction between treatment, task and activity for HR, also indicated in a *post-hoc* test on HRV), a finding associated with a moderate to large effect size (HR: Cohen's *d* = 0.80; RMSSD HRV: Cohen's *d* = 0.33), and (d) regular—relative to irregular—exercisers displayed a markedly lower HR under placebo (i.e., a three-way interaction between treatment, task and activity for HR), a finding associated with a moderate effect size (Cohen's *d* = 0.48).

We show here that irregular exercisers—those participants that did not engage in 30 min of vigorous physical activity at least 3 days a week—reported feeling more stressed after the stress task and following an acute dose of escitalopram treatment, they displayed improvements in vagally mediated cardiovascular function during stress. Importantly, these subjective and objective findings relating to the impact of acutely administered escitalopram during stress were also observed regardless of physical activity grouping. The interactions with physical activity simply highlight that the beneficial effects of escitalopram were greatest in irregular exercisers. This effect was most prominent for HR, but was also observed in planned comparisons for HR variability. The impact of escitalopram on HRV under stress was not as robust as the observed findings for HR, however, this observation is understandable because under conditions of stress, parasympathetic activity, which is the primary driver of changes in HRV, is withdrawn and this reduces the capacity of other factors to moderate reductions in HRV during stress (observed as a main effect of task).

While animal and human studies (Babyak et al., 2000; Engesser-Cesar et al., 2007; Arunrut et al., 2009) have indicated that the combination of exercise and antidepressant medication may confer no advantage over either treatment alone, there is another issue at stake here. Although antidepressant medication—including the tricyclic medications, the serotonin and noradrenaline reuptake inhibitor and the selective serotonin reuptake inhibitors—may have short- to medium-term benefits including the amelioration of depressive symptoms and increased resilience to stress, research has begun to highlight the longer-term adverse cardiovascular effects of antidepressants (Whang et al., 2009; Licht et al., 2010a). Reductions in HRV have been attributable to the specific effects of antidepressants (Licht et al., 2008, 2010a) while increases in HRV are associated with their cessation (Licht et al., 2010a). An epidemiological study (Whang et al., 2009) on 63,469 women aged 30–55 years without baseline coronary heart disease (CHD) reported that while depressive symptoms were associated with fatal CHD, antidepressant use (61% of participants were using an SSRI) was specifically associated with a 3.34 increased risk for sudden cardiac death even after controlling for a variety of confounds. A more recent study has reported that reduced cardiovascular fitness is associated with an increased risk of suicide over a 42 year follow-up period (Åberg et al., 2013) highlighting the importance of cardiovascular health over the lifespan. Together, these findings highlight the importance of research on the cardiovascular effects of antidepressants and specific lifestyle factors such as physical activity. The present study makes an important contribution to this effort highlighting that the cardiovascular effects of regular vigorous exercise and of an acute dose of escitalopram during stress are comparable, i.e., both are associated with a decrease in HR and an increase in HRV.

**Figure 3:**
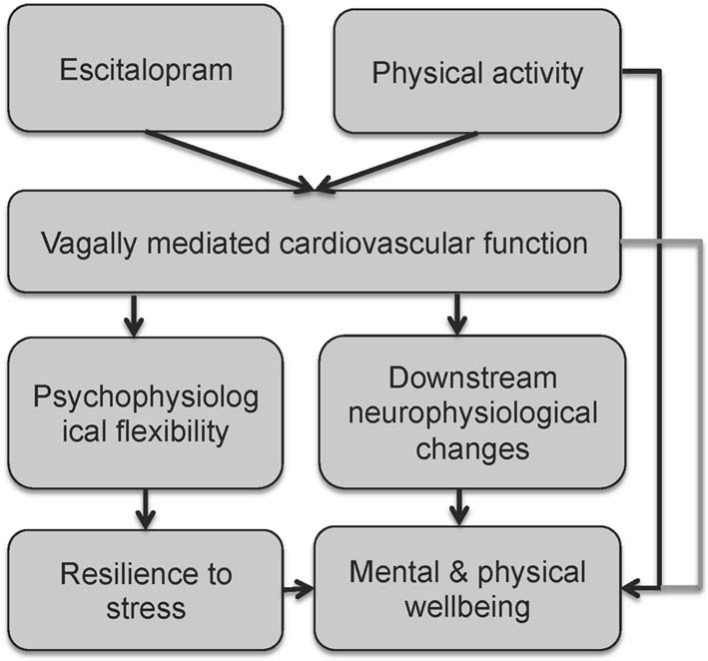
**A simplified model for the beneficial effects of regular vigorous physical activity and SSRI treatment on vagal tone, initiating a path towards mental and physical wellbeing**.

Here we propose a simplified model for the beneficial effects of regular vigorous physical activity and SSRI treatment on vagal tone, initiating a path toward mental and physical wellbeing (see Figure 3). Increased vagal function is associated with increased psychophysiological flexibility (Thayer et al., 2009; Kashdan and Rottenberg, 2010) and stress resilience, which in turn promotes wellbeing (Oveis et al., 2009; Geisler et al., 2010, 2013; Kashdan and Rottenberg, 2010; Kok and Fredrickson, 2010; Thayer et al., 2010b; Kemp and Quintana, 2013). Increased vagal function also initiates downstream neurophysiological changes such as enhanced growth factors (Follesa et al., 2007), improved regulation of HPA axis function and inflammatory processes (Tracey, 2002; Thayer and Sternberg, 2006; Huston and Tracey, 2010; Pavlov and Tracey, 2012), leading to improved wellbeing (Thayer et al., 2010b; Kemp and Quintana, 2013). The model also highlights the direct links between regular vigorous physical activity and wellbeing (discussed above). However, it is noted that over-trained athletes also display a decease in HRV, which limits the autonomic resources available to respond to emotional and physical stress (Hynynen et al., 2008; Boullosa et al., 2012).

In the present study we demonstrated that acute administration of escitalopram is associated with increased vagal function under both resting and task conditions; a finding that was particularly robust for HR. This finding is consistent with reported short- to medium-term beneficial effects of SSRIs on cardiovascular and neuroendocrine responses to stress in depressed patients (e.g., Straneva-Meuse, 2004b), but contrast against longer-term research outcomes (Whang et al., 2009; Licht et al., 2010a) highlighting the adverse effects of the SSRI class of antidepressants. Here, an explanation for this apparent discrepancy is the complexity of central and autonomic 5-HT effects on cardiovascular function, which include bradycardia, associated with activation of 5-HT_1A_ receptors, as well as tachycardia, associated with activation of 5HT_2_ receptors. It is possible that the effects of SSRIs shift from parasympathetic—as shown here—to sympathetic activation with increasing length of use. Further research is urgently needed on the combination of antidepressants and physical activity over longer-time frames than typical clinical trials (i.e., years rather than months).

A number of limitations are worth noting. Participants correctly guessed treatment condition beyond chance due to side effects, consistent with those typically experienced with escitalopram (Cipriani et al., 2009b). However, neither experience of side effects, nor accuracy of treatment guess impacted on HR or HRV. Experimenters were also blinded to individual participants' physical activity levels and participants were not aware that physical activity was of primary interest. It is therefore unlikely that the experience of side effects and correctly guessing treatment would have confounded the obtained findings. Another limitation is the use of a questionnaire based-measure of physical activity. It is possible that our measure reflects recent regular physical activity, rather than fitness *per se*. Future studies could consider use of objective measures of fitness such as VO_2_ max to confirm that those engaging in regular activity are indeed characterized by higher levels of fitness. Future studies with clinical samples and chronic treatment are also needed to further examine the utility of combining antidepressant medication and vigorous physical activity.

In summary, results from this experimental study highlight the adverse impact of stress on vagally mediated cardiovascular function and the beneficial moderating effects of escitalopram as well as regular vigorous physical exercise. Escitalopram was observed to increase vagal function under both rest and stress conditions, and under stress, these effects are specific to irregular exercisers, but not regular exercisers. We propose a simplified model for understanding the beneficial effects of physical activity and antidepressants on cardiovascular function that may subsequently lead to improved mental and physical activity. Our study and model provide a foundation on which future research could be based. Increasing concerns about the bidirectional relationship between depression and CVD, reinforce the need for further research on the impact of antidepressants, physical activity and their combination, that is not only focused on amelioration of depressive symptoms, but also on vagally mediated cardiovascular function.

## Disclosure

This research was supported by an Australian Research Council Discovery Project Grant (DP0987332), a National Health and Medical Research Council (NHMRC) Project Grant (464863) and a NHMRC Career Development Award (571101) awarded to Andrew H. Kemp. The authors Andrew H. Kemp and Tim Outhred are currently supported by an International Visiting Research Professorship from the Universidade de São Paulo and an Australian Postgraduate Award, respectively. Camilla S Hanson was supported by a Charles Perkins Centre Summer Research Scholarship.

### Conflict of interest statement

Gin S. Malhi has received research support from AstraZeneca, Eli Lilly, Organon, Pfizer, Servier, and Wyeth. He has been a speaker for AstraZeneca, Eli Lilly, Janssen Cilag, Lundbeck, Pfizer, Ranbaxy, Servier, and Wyeth. He has been a consultant for AstraZeneca, Eli Lilly, Janssen Cilag, Lundbeck, and Servier. Andre R. Brunoni declares no potential conflict of interest.
